# Supplementary material for: Mitotic bookmarking redundancy by nuclear receptors in pluripotent cells
Source: Nat Struct Mol Biol. 2024 Jan 9;31(3):513–22. doi: 10.1038/s41594-023-01195-1 (PMC10948359; doi:10.1038/s41594-023-01195-1)

Extended Data Fig.1A

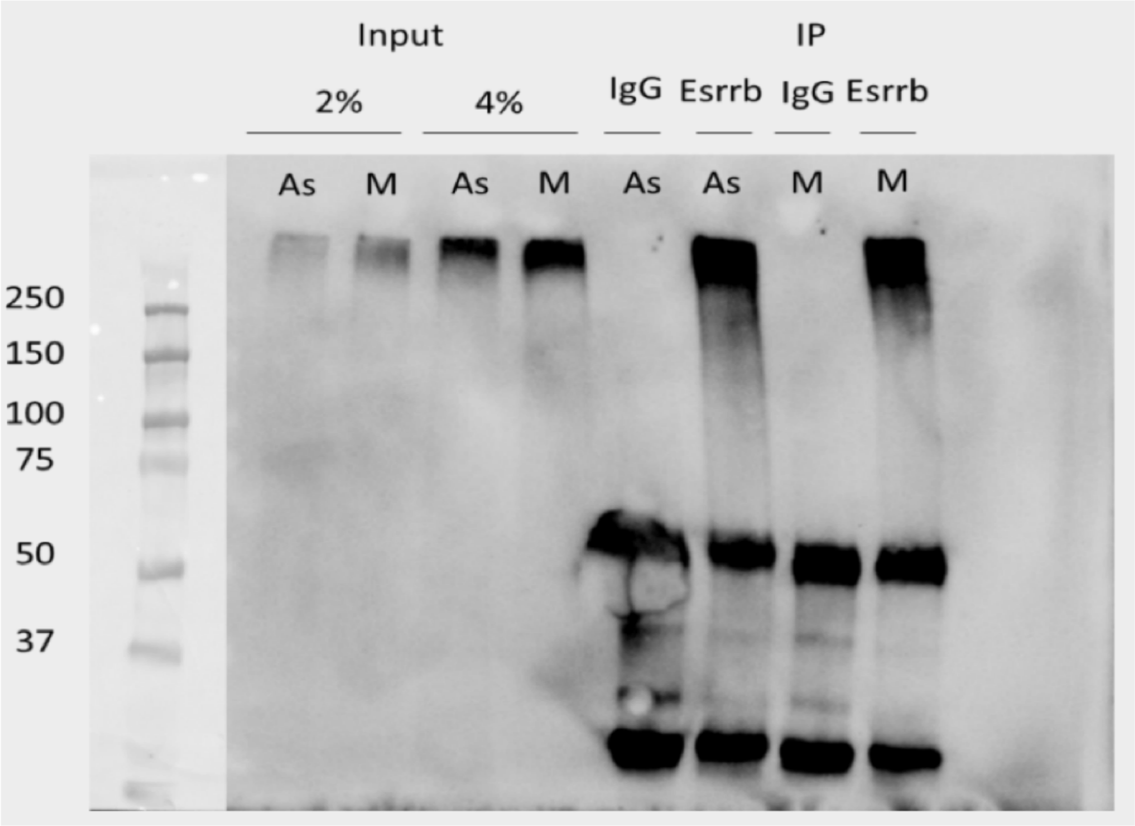

Extended Data Fig.7F

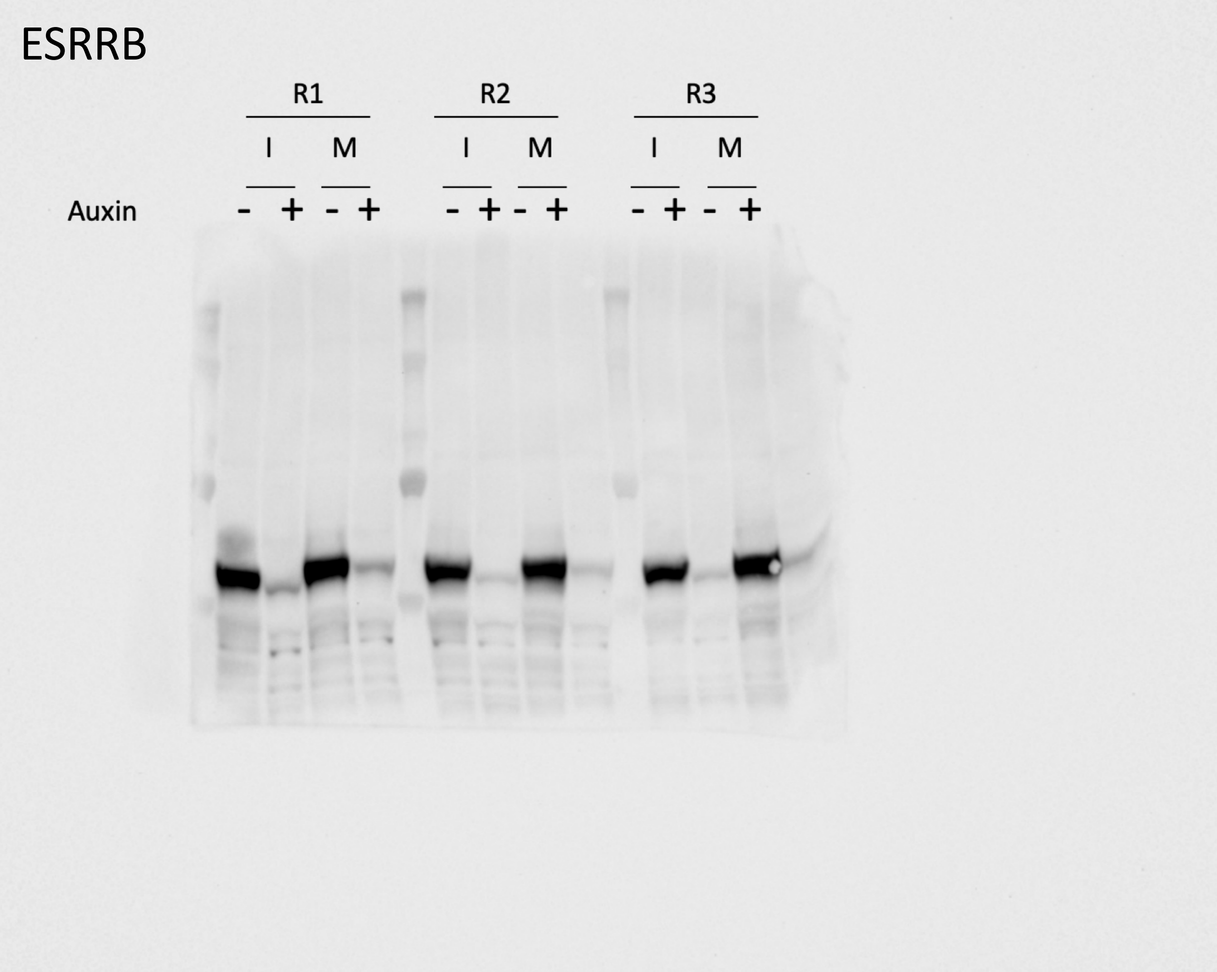

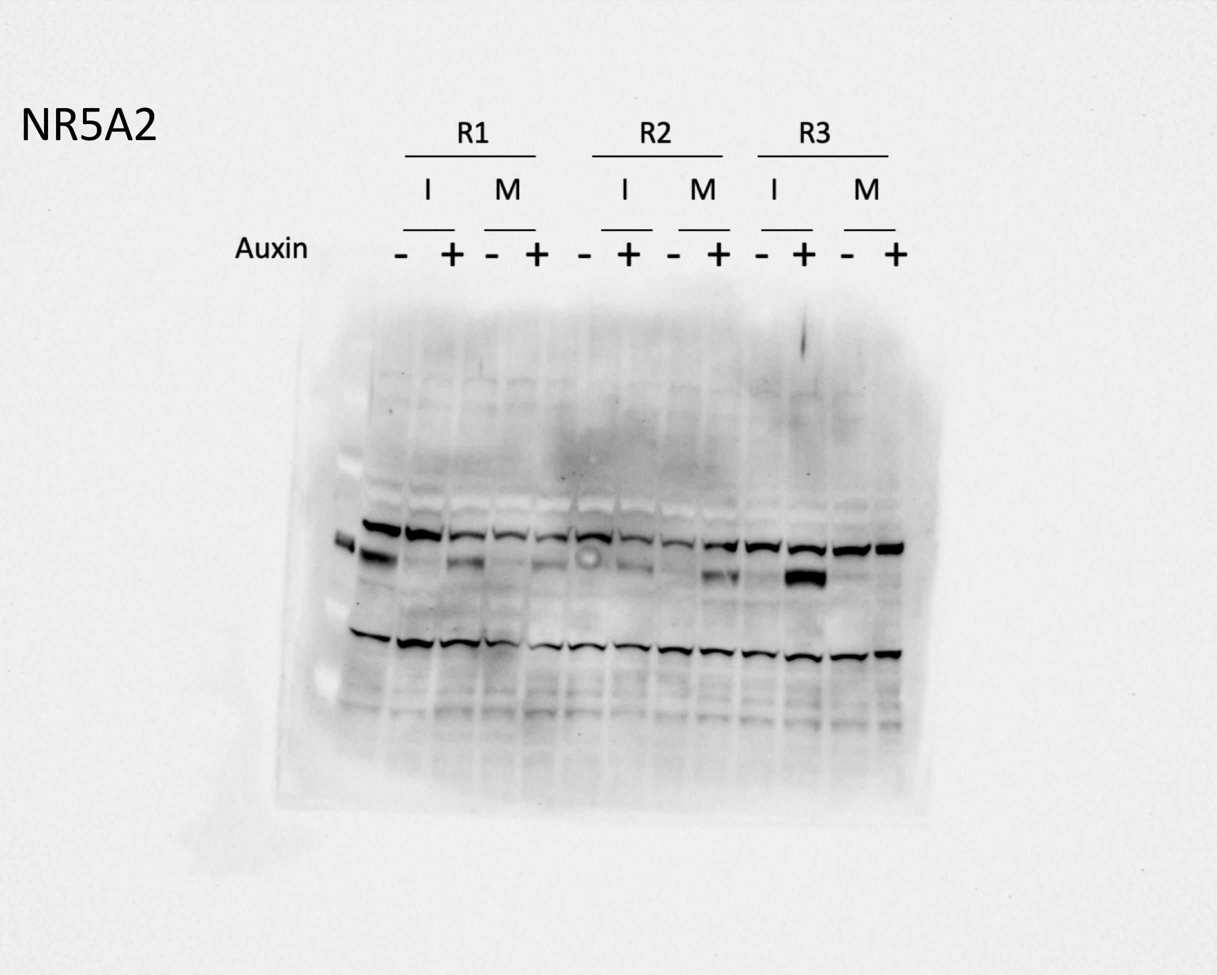

Extended Data Fig.7D

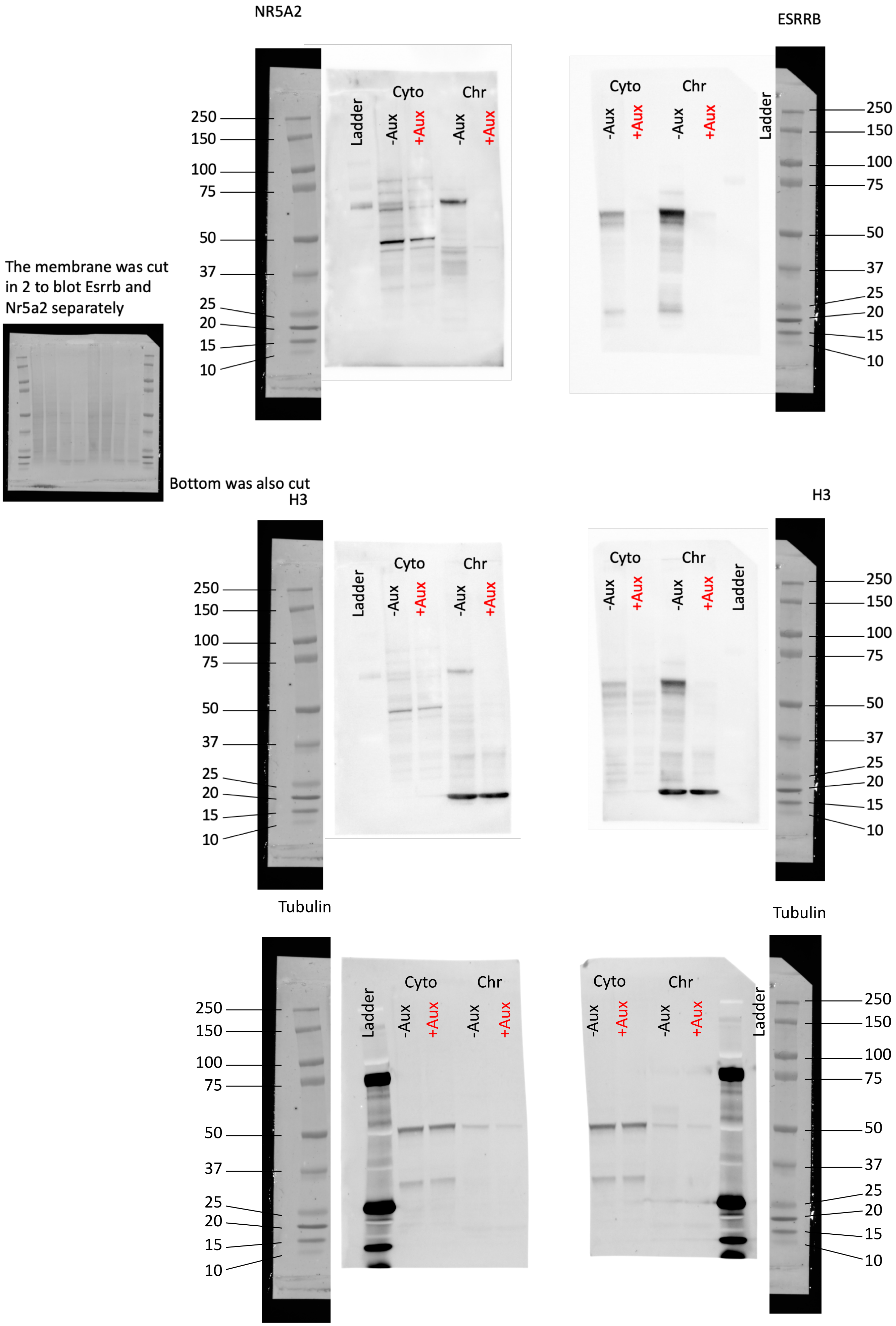

Supplement: Supplementary file 4 — Unprocessed western blots. [file 41594_2023_1195_MOESM4_ESM.pdf]
